# MYC Rearranged Aggressive B-Cell Lymphomas: A Report on 100 Patients of the Fondazione Italiana Linfomi (FIL)

**DOI:** 10.1097/HS9.0000000000000305

**Published:** 2019-11-09

**Authors:** Maria Chiara Tisi, Simone Ferrero, Irene Dogliotti, Cristina Tecchio, Giuseppe Carli, Mattia Novo, Piero Maria Stefani, Sara Rattotti, Monica Balzarotti, Dario Marino, Matteo Pelosini, Alessandra Romano, Leonardo Flenghi, Vittorio Ruggero Zilioli, Teresa Calimeri, Arianna Di Napoli, Manuela Zanni, Erica Finolezzi, Federico Mosna, Guido Gini, Giovanna Mansueto, Alice Di Rocco, Gabriella Tomei, Nicola Sgherza, Jacopo Olivieri, Luca Nassi, Francesco Piazza, Angelo Fama, Antonio Greco, Margherita Giannoccaro, Anna Maria Mazzone, Carlo Visco, Giacomo Loseto, Francesco Zaja

**Affiliations:** 1 Cell Therapy and Hematology, San Bortolo Hospital, Vicenza, Italy; 2 Institute of Hematology, Catholic University of the Sacred Heart, Rome, Italy; 3 Division of Hematology 1, Department of Molecular Biotechnologies and Health Sciences, University of Torino/AOU “Città della Salute e della Scienza di Torino”, Torino, Italy; 4 Medicine, Section of Hematology and Bone Marrow Transplant Unit, University of Verona, Verona, Italy; 5 Division of Hematology 2, AOU “Città della Salute e della Scienza di Torino”, Torino, Italy; 6 HEMATOLOGY UNIT Department of Medicine, General Hospital TREVISO, Italy; 7 Division of Hematology, Fondazione IRCCS Policlinico San Matteo, Pavia, Italy; 8 Hematology Unit, Humanitas Clinical and Research Center – IRCCS - Rozzano/Milano, Italy; 9 Medical Oncology 1, Veneto Institute of Oncology IOV IRCCS, Padova, Italy; 10 Clinical and Experimental Medicine, Section of Hematology, University of Pisa, Pisa, Italy; 11 Hematology, AOU Policlinico Vittorio Emanuele, Catania, Italy; 12 Hematology, O. Santa Maria della Misericordia, Perugia, Italy; 13 Division of Hematology, ASST Grande Ospedale Metropolitano Niguarda, Milan, Italy; 14 Lymphoma Unit, Department of Onco-Hematology, IRCCS San Raffaele Scientific Institute, Milan, Italy; 15 Department of Clinical and Molecular Medicine, Roma Sapienza University, Sant’Andrea Hospital, Rome, Italy; 16 Hematology, AO SS Antonio e Biagio, Alessandria, Italy; 17 Hematology, Transfusional Medicine and Biotechnologies, UOSD “Centro Diagnosi e Terapia dei Linfomi”, PO Santo Spirito, Pescara, Italy; 18 Hematology and BMT Unit, Ospedale Centrale San Maurizio, ASDAA, Bolzano, Italy; 19 Hematology Clinic, Department of Clinical and Molecular Sciences, Marche Polytechnic University, Ancona, Italy; 20 Hematology and Stem Cell Transplantation Unit, IRCCS-CROB, Referral Cancer Center of Basilicata, Rionero in Vulture, PZ, Italy; 21 Cellular Biotechnologies and Hematology, 'Sapienza University’, Rome, Italy; 22 Hematology, Ospedale di Ivrea-ASL TO4, Ivrea, Italy; 23 Hematology, IRCCS Ospedale Casa Sollievo Sofferenza, S. G. Rotondo, Italy; 24 Hematology, Ospedale di Civitanova Marche, Civitanova Marche, Italy; 25 Clinica Ematologica, Centro Trapianti e Terapie Cellulari “Carlo Melzi”, Azienda Sanitaria Universitaria Integrata di Udine, Italy; 26 SCDU Ematologia, AOU Maggiore della Carità, Novara, Italy; 27 Hematology, University of Padova, Padova, Italy; 28 Hematology, Azienda Unità Sanitaria Locale di Reggio Emilia – IRCCS, Reggio Emilia, Italy; 29 Hematology and BM transplant unit A.O.C. Panico Tricase (Le), Italy; 30 Hematology, ASL Le/1 P.O. Vito Fazzi, Lecce, Italy; 31 Hematology, ASL Taranto, Taranto, Italy; 32 Department of Medicine, Section Hematology, University of Verona, Italy; 33 Hematology and Cell Therapy Unit, IRCCS Istituto Tumori “Giovanni Paolo II”, Bari, Italy; 34 Hematology, Azienda Sanitaria Universitaria Integrata Trieste, Italy.

Advances in the classification of diffuse large B cell lymphoma (DLBCL) based on genetic aberrations and molecular expression profiles have led to the recognition of a new category of high grade B cell lymphoma, including “high grade B-cell lymphoma with rearrangements of MYC and BCL2 and/or BCL6”, in the 2016 revised 4th edition of World Health Organization (WHO) Classification of Tumors of Haematopoietic and Lymphoid Tissues.^1^

The poor results achieved with R-CHOP (Rituximab, Cyclophosphamide, Doxorubicin, Vincristine and Prednisone) in multiple studies, have prompted the use of intensive treatments, such as Burkitt lymphoma regimens, stem-cell transplantation or dose-adjusted EPOCH-R (DA-EPOCH-R)^2–6^ as first line treatments, even if no prospective comparisons are available.

On these bases we performed a “real life” retrospective multicenter study with the aim of characterizing the clinical and pathological landscape of MYC rearranged aggressive B-cell lymphomas in Italy, and comparing for efficacy the preferred therapeutic choices.

In the years 2011 to 2017, a total of 100 patients with newly diagnosed DLBCL (n = 65) or B cell lymphoma, unclassifiable (BCLU, n = 35) were collected from 29 Italian centers of the Fondazione Italiana Linfomi (FIL) Fig. S1 (Supplemental Digital Content). Pathological data were retrieved from centrally reviewed local pathological reports. FISH (*fluorescent in-situ hybridization)* analysis was performed with current standard methods.^7^

Treatment groups were classified as follows: 1) “intensive regimens”: incorporating high dose methotrexate: R-CODOX-M/IVAC [Rituximab, Cyclophosphamide, Oncovin (Vincristine), Doxorubicin, Methotrexate alternating with Rituximab, Ifosfamide, Vepesid (etoposide), Ara-C (Cytarabine)], n = 23, GMALL-R (German Multicenter Study Group for the Treatment of Adult Acute Lymphoblastic Leukemia, Rituximab), n = 9, R-Hyper-CVAD/R-MA [Rituximab, Hyperfractionated Cyclophosphamide, Vincristine, Adriamycin (Doxorubicin), Dexamethasone alternating with Rituximab, Methotrexate, Ara-C (Cytarabine)], n = 10; 2) “R-CHOP-like”: including also R-COMP, n = 6 (R-CHOP with liposomal anthracycline), R-miniCHOP, n = 2, R-M/VACOP-B (Rituximab, Methotrexate/Etoposide, Doxorubicin, Cyclophosphamide, Vincristine, Prednisone, and Bleomycin), n = 2, R-mega-CHOP, n = 1; 3) “DA-EPOCH-R”: dose-Adjusted Etoposide, Doxorubicin, Cyclophosphamide, Vincristine, Prednisone and Rituximab. Patients treated with different approaches or palliative care (“others”) were censored for survival analysis, or analyzed separately.

The primary end-point was progression-free survival (PFS), defined as the time between lymphoma diagnosis and relapse or progression, lack of response, or death from any cause. Survival curves were estimated using the Kaplan-Meier product limit method. Statistical analyses were performed using the Stata 13.0 software (Stata Corp., College Station, TX).

All 100 patients presented MYC rearrangement as for inclusion criteria, 57 patients were double hit for BCL2 (DHL-BCL2) and 29 were double hit for BCL6 (DHL-BCL6). The remaining 19 MYC rearranged patients, lacking an additional BCL2 or BCL6 translocation, were defined single hit lymphomas (SHL – MYC+/BCL2-/BCL6-). Five patients carried all the three rearrangements, so called “triple hit lymphoma” (THL **–** MYC+/BCL2+/BCL6+) and for survival analysis were considered among DHLs.

Clinical and pathological features of the whole series are detailed in Table 1. Eight patients had central nervous system (CNS) involvement at diagnosis (2 parenchymal, 6 meningeal).

**Table 1:**
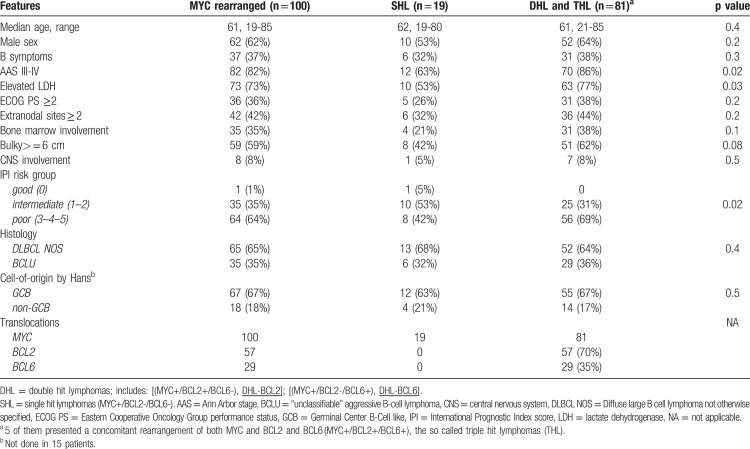
Patient and Tumor Characteristics.

The majority of patients (n = 42) were treated upfront with intensive schedules, followed by R-CHOP like (n = 35) and by DA-EPOCH-R (n = 18). Five patients were treated with other schemes (Supplementary Table S1, Supplemental Digital Content). Eighteen patients received ASCT consolidation. Overall, 70 patients received CNS prophylaxis: intrathecal in 24, systemic MTX in 8, and both in 38. Finally, 20 patients received radiotherapy consolidation. Patients treated with intensive regimens presented with similar baseline features to patients treated with R-CHOP, with exception of more advanced IPI in intensive group (p = 0.006), and similar to DA-EPOCH-R group, with the exception of higher ki67 values (p = 0.04), and advanced stage (p = 0.02). Finally, patients presenting a BCLU histology received more frequently intensive treatment than standard R-CHOP or DA-EPOCH-R, p = 0.001 and p = 0.05, respectively, (Supplementary Table S2, Supplemental Digital Content).

Overall response was 71%, with 58 patients achieving complete response (CR) and 13 partial response (PR) at the end of front-line treatment. Three patients died of treatment related complications (infections) during induction, and one did not receive any treatment. CR rate was 68% in SHL and 56% in DHL (p = 0.2), see Supplementary Table S2 (Supplemental Digital Content). Patients treated with intensive schedules had a CR of 69% (29/42), which was not statistically different from patients treated with R-CHOP (18/35, 51%, p = 0.08), and similar to patients treated with DA-EPOCH-R (11/18, 61%, p = 0.4).

After a median follow up for survivors of 33 months, the 24 months PFS and OS of the overall MYC rearranged (MYC-R) population were 54% (95% CI 44–63) and 57% (95% CI 46–66), respectively, Fig. S2A and Fig. S2B (Supplemental Digital Content). For the 19 patients with SHL and the 81 patients with DHL, PFS at 24 months was 77% (95% CI 51–91) and 49% (95% CI 37–59), respectively (p = 0.05), Fig. S2C (Supplemental Digital Content), and OS was 77% (95% CI 50–90) vs 52% (95% CI 40–63), respectively (p = 0.1), Fig. S2D. PFS did not vary significantly based on the specific double rearrangements found (ie, DHL-BCL2 vs DHL-BCL6 vs THL), Fig. S3 (Supplemental Digital Content).

Analyzing all the MYC-R population, the 24 months PFS was 59% (95% CI 33–78) in patients treated with DA-EPOCH-R (p = 0.6, compared with intensive treatments), 46% (95% CI 29–62) in those receiving R-CHOP-like schedules (p = 0.04), and 65% (95% CI 49–68) in patients who received intensive treatments, Fig. 1. In the same population, the 24 months OS was 74% (95% CI 45–89) in patients treated with DA-EPOCH-R, which was superior to patients receiving R-CHOP-like schedules, 46% (95% CI 27–62), p = 0.05. However, no significant OS difference was seen between DA-EPOCH-R and intensive regimens. Moreover, restricting the analysis to the group of elderly patients (age > 65 years, n = 42), no survival advantage was observed in patients treated with DA-EPOCH-R or intensive regimens vs R-CHOP-like. Superimposable results were obtained selecting the 81 DHL patients only, with a PFS and OS advantage for patients receiving DA-EPOCH-R and intensive regimens vs R-CHOP-like (p = 0.02), Fig. S4 (Supplemental Digital Content). 24 months OS in the 81 DHL patients was 66% (95% CI 33–86) for DA-EPOCH-R, 34% (95% CI 15–53) for R-CHOP-like (p = 0.03 and p = 0.05 respectively), and 64% (95% CI 46–77) for intensive treatments.

**Figure 1:**
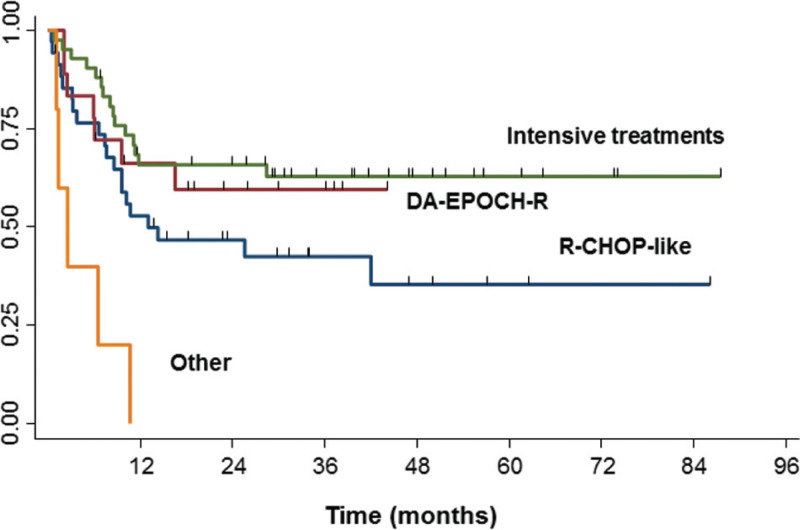
PFS by delivered treatment: 24 months PFS 65% (95% CI 49–68) in patients who received intensive treatments, 46% (95% CI 29–62) in those who received R-CHOP-like schedules (p = 0.04 compared with intensive and DA-EPOCH-R); 59% (95% CI 33–78) in patients treated with DA-EPOCH-R (p = 0.6, compared with intensive treatments) and not evaluable in patients treated with other treatments (p = 0.001).

ASCT consolidation (n = 18) did not give any PFS advantage in patients achieving CR or PR at the end of induction treatment (p = 0.3 and 0.4, respectively), Fig. S5A and Fig. S5B (Supplemental Digital Content). Actually, patients in CR at the end of induction retained a good 24 months PFS even without ASCT (82%–95% CI 69–90).

Moreover, despite quite aggressive CNS prophylaxis, 5/100 patients experienced a CNS relapse during observation period (two of them presenting a CNS involvement already at diagnosis). Finally, the outcome after relapse was very poor, accounting for a 24 months OS of 13% (95% CI 4–24), Fig. S6 (Supplemental Digital Content).

In the overall MYC-R population, B-symptoms (p = 0.05), ECOG performance score (PS) > 2 (p = 0.003), bone marrow infiltration (p = 0.005), elevated LDH levels (p = 0.03) and poor risk IPIaa (p = 0.05), predicted worse PFS (Supplementary Table S3, Supplemental Digital Content). Restricting analysis for DHL population, only ECOG PS > 2 (p = 0.01) and bone marrow infiltration (p = 0.05) retained their prognostic impact. Finally, after Cox proportional hazard multivariate analysis, only a poor ECOG (> 2) remained predictive of poor PFS, p = 0.009 (95% CI 1.21–3.86), HR 2.16.

Overall, the main results of this study can be summarized as follows:

1) “High-grade B-cell lymphoma with MYC and BCL2/BCL6 rearrangements” show an overall worse prognosis than “single-hit” MYC-R patients in terms of PFS;
2) frontline intensive chemotherapy and DA-EPOCH-R are associated with a significant survival benefit if compared with R-CHOP-like treatments;
3) restricting the analysis to the group of elderly patients, the advantage for intensive regimens was no longer observed, suggesting that younger patients might benefit the most from these schedules;
4) survival is not significantly impacted by ASCT consolidation, regardless of the clinical response observed;
5) survival after progression in “high-grade B-cell lymphoma with MYC and BCL2/BCL6 rearrangements” is particularly dismal, suggesting that salvage therapies are ineffective in this setting.

Therefore, with the limits of a retrospective series, we reported a large, National, homogeneous population, where the distribution of the baseline characteristics and the clinical results are very similar to those of previously published unselected cohorts^2,8,9^ As regards CNS relapse, its cumulative incidence was inferior to that of the MDACC retrospective cohort^8^ (5% at 2 years vs 13% at 3 years). Even if the numbers are too small to drive sound correlations between the received prophylaxis (intrathecal vs systemic) and the characteristics of CNS relapse (meningeal vs parenchimal), this difference might be due to a more aggressive upfront CNS prophylaxis in our series. Finally, as screening of all DLBCL and BCLU cases for MYC rearrangement was not the routine practice of all participating centers, we cannot exclude the possibility that selection bias influenced the decision to test specific cases.

In conclusion, the results of our study confirm that R-CHOP is not an appropriate therapeutic choice for “high-grade B-cell lymphoma with MYC and BCL2/BCL6 rearrangements”, and that intensive regimens or DA-EPOCH-R should be preferred, especially for younger patients. Nevertheless, the current diagnostic work-up of aggressive B cell lymphomas is controversial, and the screening strategy to drive FISH testing is not clearly shared.^10–12^. Moreover, the complex biological landscape of DLBCL probably cannot be simply resumed by classical FISH or cell of origin analysis: actually, new biological insights, including high-throughput mutational^13,14^ and microenvironmental studies,^15^ are needed for a better classification and a more effective testing of front line targeted agents.

## Supplementary Material

Supplemental Digital Content

## References

[1] Swerdlow SH Campo E Pileri SA The 2016 revision of the World Health Organization classification of lymphoid neoplasms. *Blood.* 2016;127:2375–2390. 26980727 10.1182/blood-2016-01-643569 PMC4874220

[2] Petrich AM Gandhi M Jovanovic B Impact of induction regimen and stem cell transplantation on outcomes in double-hit lymphoma: a multicenter retrospective analysis. *Blood.* 2014;124:2354–2361. 25161267 10.1182/blood-2014-05-578963

[3] McPhail ED Maurer MJ Macon WR Inferior survival in high-grade B-cell lymphoma with MYC and BCL2 and/or BCL6 rearrangements is not associated with MYC/IG gene rearrangements. *Haematologica.* 2018;103:1899–1907. 29903764 10.3324/haematol.2018.190157 PMC6278976

[4] Dunleavy K Fanale MA Abramson JS Dose-adjusted EPOCH-R (etoposide, prednisone, vincristine, cyclophosphamide, doxorubicin, and rituximab) in untreated aggressive diffuse large B-cell lymphoma with MYC rearrangement: a prospective, multicentre, single-arm phase 2 study. *Lancet Haematol.* 2018;5:e609–e617. 30501868 10.1016/S2352-3026(18)30177-7 PMC6342507

[5] Howlett C Snedecor SJ Landsburg DJ Front-line, dose-escalated immunochemotherapy is associated with a significant progression-free survival advantage in patients with double-hit lymphomas: a systematic review and meta-analysis. *Br J Haematol.* 2015;170:504–514. 25907897 10.1111/bjh.13463

[6] Visco C Tzankov A Xu-Monette ZY Patients with diffuse large B-cell lymphoma of germinal center origin with BCL2 translocations have poor outcome, irrespective of MYC status: a report from an International DLBCL rituximab-CHOP Consortium Program Study. *Haematologica.* 2013;98:255–263. 22929980 10.3324/haematol.2012.066209 PMC3561433

[7] Tzankov A Xu-Monette ZY Gerhard M Rearrangements of MYC gene facilitate risk stratification in diffuse large B-cell lymphoma patients treated with rituximab-CHOP. *Mod Pathol.* 2014;27:958–971. 24336156 10.1038/modpathol.2013.214

[8] Oki Y Noorani M Lin P Double hit lymphoma: the MD Anderson Cancer Center clinical experience. *Br J Haematol.* 2014;166:891–901. 24943107 10.1111/bjh.12982

[9] Johnson NA Savage KJ Ludkovski O Lymphomas with concurrent BCL2 and MYC translocations: the critical factors associated with survival. *Blood.* 2009;114:2273–2279. 19597184 10.1182/blood-2009-03-212191 PMC2745846

[10] Scott DW King RL Staiger AM High-grade B-cell lymphoma with MYC and BCL2 and/or BCL6 rearrangements with diffuse large B-cell lymphoma morphology. *Blood.* 2018;131:2060–2064. 29475959 10.1182/blood-2017-12-820605 PMC6158813

[11] Copie-Bergman C Double-hit DLBCL: should we limit FISH testing? *Blood.* 2018;131:1997–1998. 29724715 10.1182/blood-2018-03-836361

[12] Ennishi D Jiang A Boyle M Double-hit gene expression signature defines a distinct subgroup of germinal center B-Cell-like diffuse large B-Cell lymphoma. *J Clin Oncol.* 2019;37:190–201. 30523716 10.1200/JCO.18.01583 PMC6804880

[13] Schmitz R Wright GW Huang DW Genetics and pathogenesis of diffuse large B-Cell lymphoma. *N Engl J Med.* 2018;378:1396–1407. 29641966 10.1056/NEJMoa1801445 PMC6010183

[14] Chapuy B Stewart C Dunford AJ Molecular subtypes of diffuse large B cell lymphoma are associated with distinct pathogenic mechanisms and outcomes. *Nat Med.* 2018;24:679–690. 29713087 10.1038/s41591-018-0016-8 PMC6613387

[15] Ciavarella S Vegliante MC Fabbri M Dissection of DLBCL microenvironment provides a gene expression-based predictor of survival applicable to formalin-fixed paraffin-embedded tissue. *Ann Oncol.* 2018;29:2363–2370. 30307529 10.1093/annonc/mdy450 PMC6311951

