# Supplementary material for: MYC Rearranged Aggressive B-Cell Lymphomas: A Report on 100 Patients of the Fondazione Italiana Linfomi (FIL)
Source: Hemasphere. 2019 Nov 9;3(6):e305. doi: 10.1097/HS9.0000000000000305 (PMC6924554; doi:10.1097/HS9.0000000000000305)
Supplement: Supplemental Digital Content [file hs9-3-e305-s001.docx]

**Supplemental Digital Content**

**Figure S1.**

Distribution of 100 MYC-R patients according to additional translocations: 57 (57%) patients were double hit for BCL2 (**DHL-BCL2**) and 29 (29%) were double hit for BCL6 (**DHL-BCL6**). The remaining 19 MYC rearranged patients, lacking an additional BCL2 or BCL6 translocation, were defined single hit lymphomas (**SHL – MYC+/BCL2-/BCL6-**). Five patients (5%) carried all the three rearrangements, so called “triple hit lymphoma” (**THL – MYC+/BCL2+/BCL6+**).

**
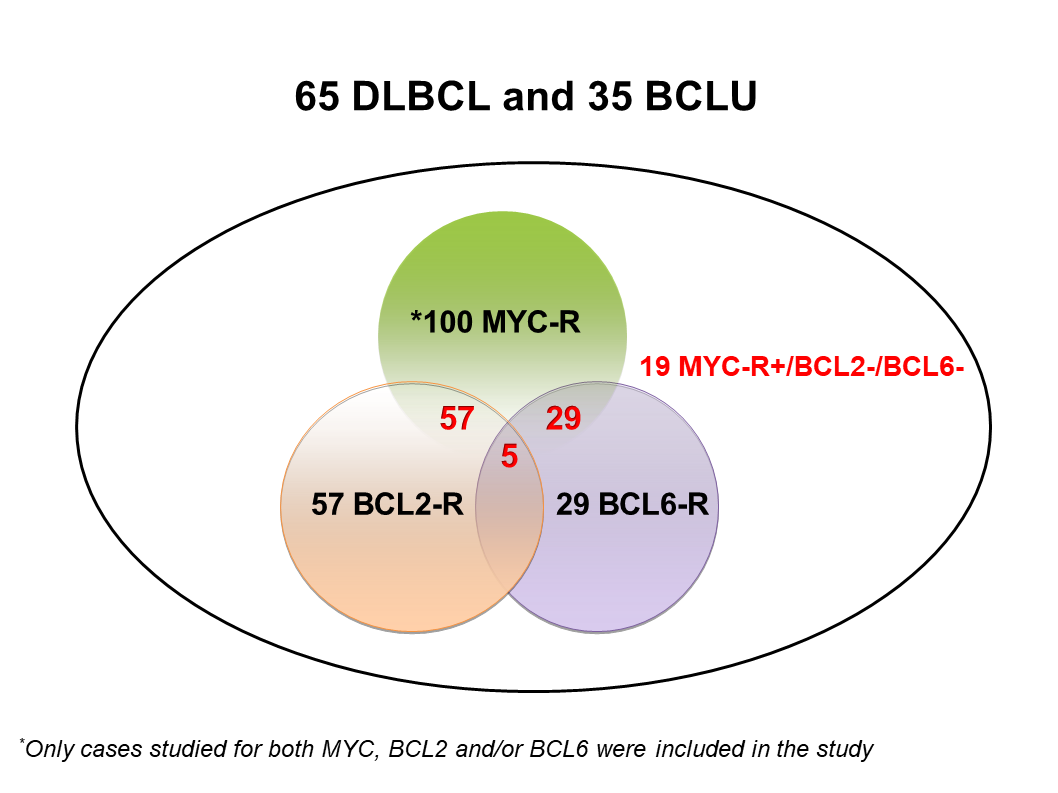
**

**Figure S2.**

Progression-free survival (PFS) and overall survival (OS) of the overall MYC rearranged population: **Fig. S2A**, 24 months PFS 54%, **Fig.** **S2B**, 24 months OS 57%.

**Fig. S2C**: 24 months PFS of the 19 patients with single hit (MYC) vs the 81 patients with double hit (DHL): 77% vs 49%, p=0.05.

**Fig. S2D**: 24 months OS for SHL 77% vs DHL 52%, p=0.1.

Triple hit lymphoma were included among DHLs for survival analysis.

**
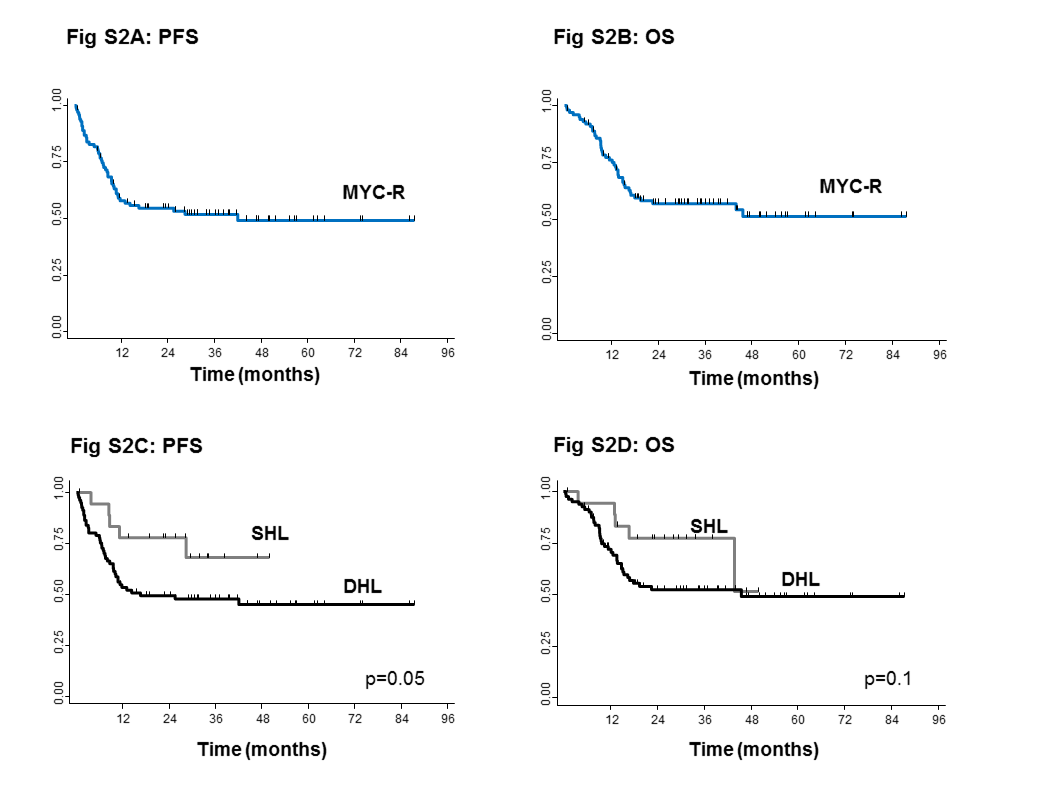
**

**Figure S3.**

PFS based on the specific double rearrangements found. **SHL**= MYC+/BCL2-/BCL6-; **THL**= MYC+/BCL2+/BCL6+; **DHL-BCL2**= double hit for MYC and BCL2; **DHL-BCL6**= double hit for MYC and BCL6; (p=0.14).

**
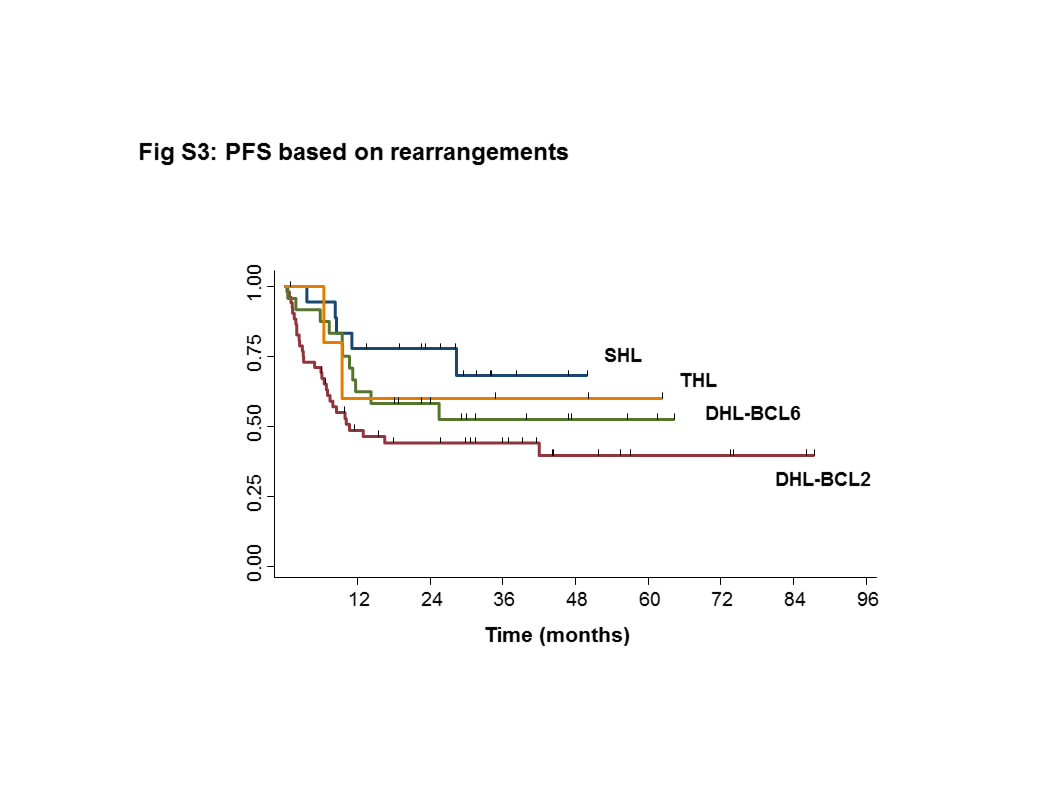
**

**Figure S4.**

PFS by delivered treatment in 81 DHL: 24 months PFS in 81 MYC-R patients was: 64% (95% CI 45-77) for intensive treatments, 40% (95% CI 22-58) for R-CHOP-like and 50% (95% CI 23-73) for DA-EPOCH-R, with a significantly advantage in terms of PFS for patients who received intensive regimens vs R-CHOP-like (p=0.02).

Triple hit lymphoma were included among DHLs for survival analysis.

**
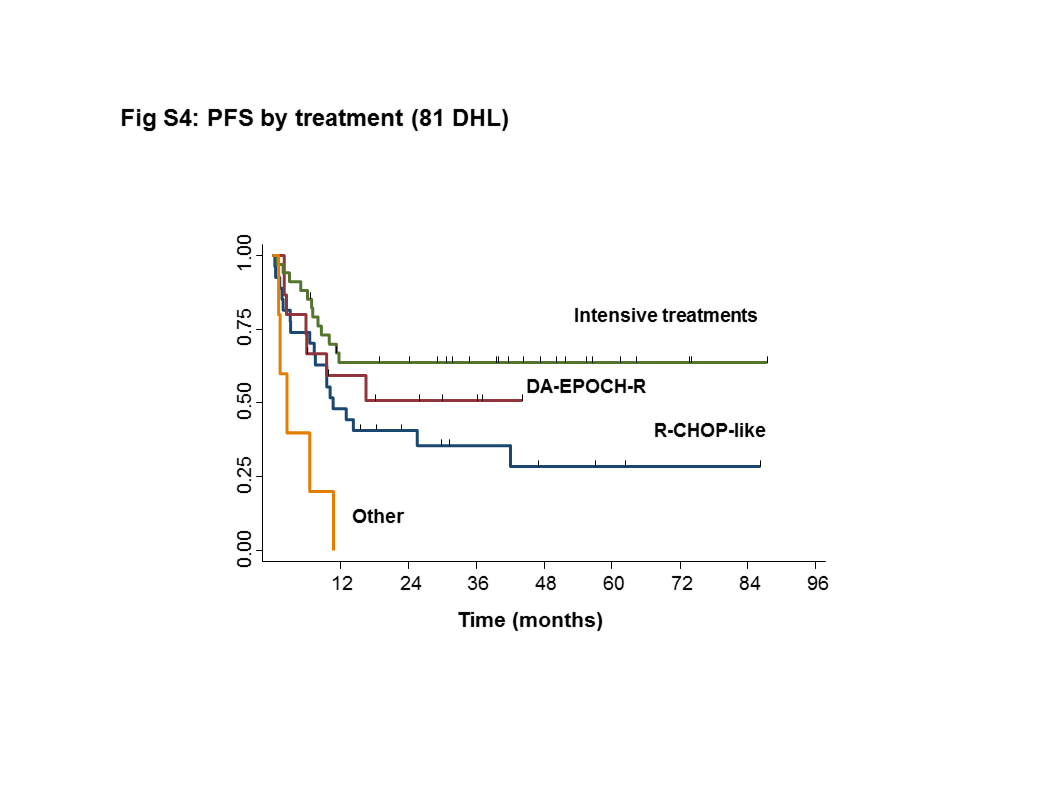
**

**Figure S5A and S5B.**

Autologous stem cell transplantation (ASCT) consolidation (n=18) did not give any PFS advantage in complete remission (CR) – **(Fig. S3A**), or partial remission (PR) patients, **(Fig. S3B)**, at the end of induction treatment (p=0.3 and 0.4, respectively).

**
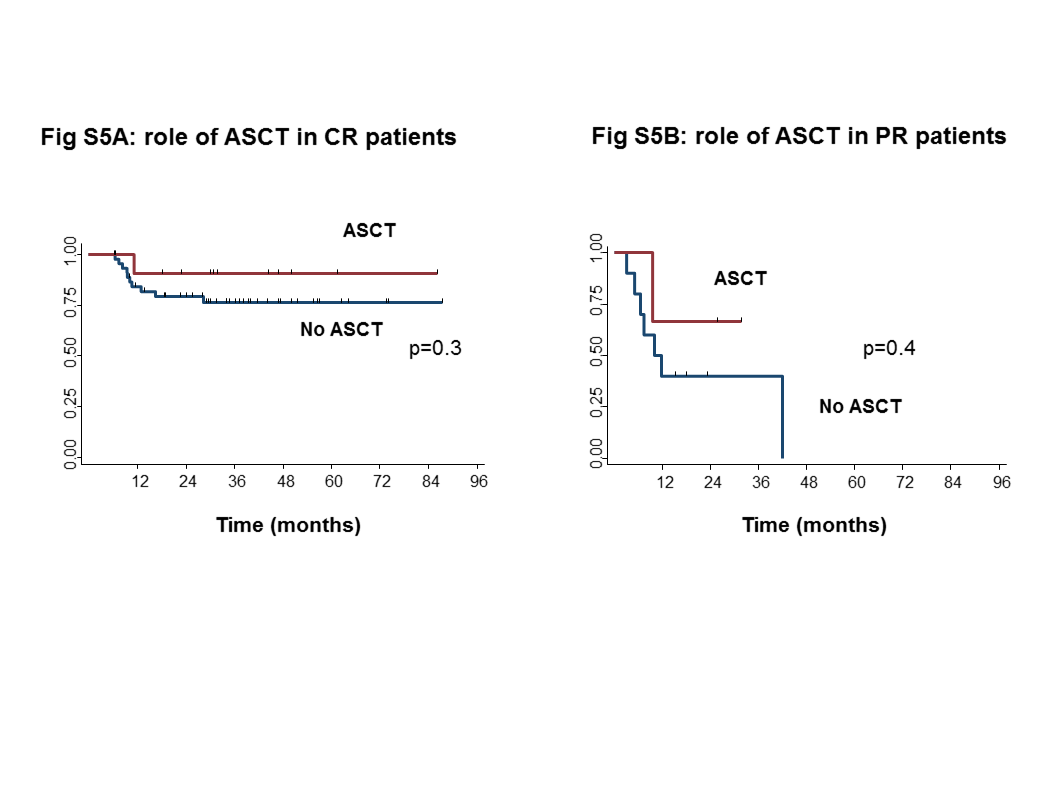
**

**Figure S6.**

OS in relapsed/refractory patients: 24 months OS calculated from the time of PFS to the last follow-up after first line therapy [13% (95% CI 0.04-0.24)].


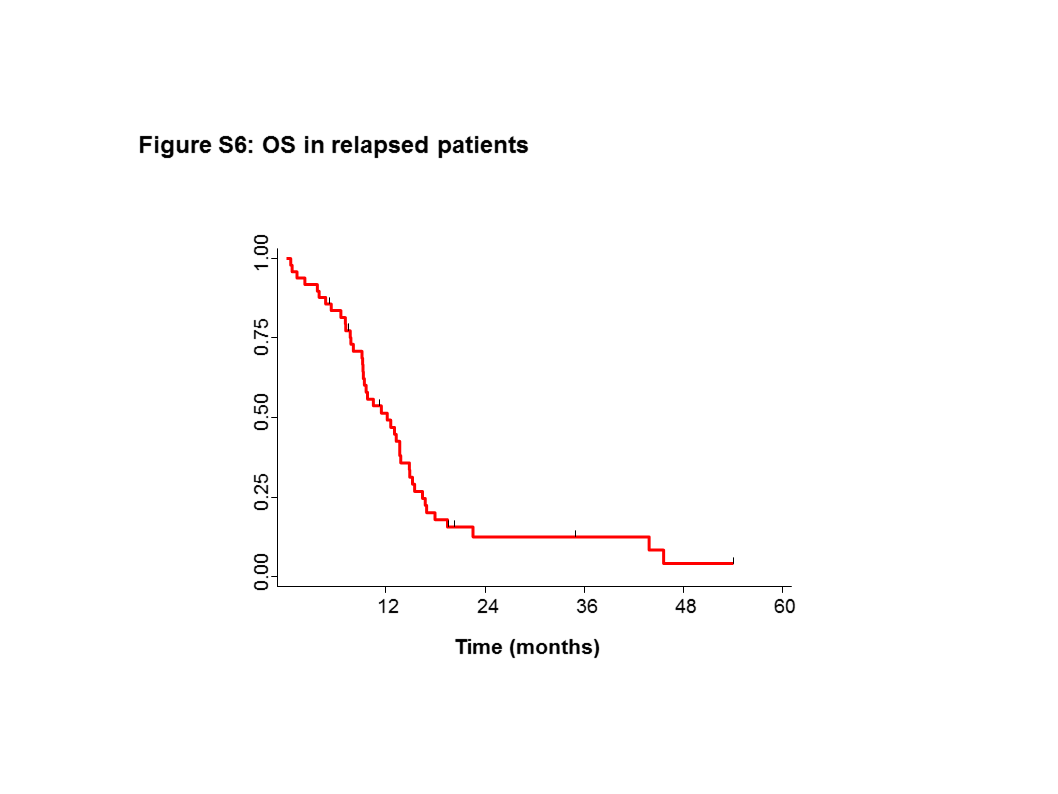


**Table S1: Treatment and response**

|  | **MYC rearranged (n=100)** | **SHL (n=19)** | **DHL and THL (n=81)** | **p value** |
| --- | --- | --- | --- | --- |
| **Total patients** | 100 | 19/100 (19%) | 81/100 (81%) |  |
|  |  |  |  |  |
| *Intensive regimens** | 42 (42%) | 8 (42%) | 34 (42%) | 1 |
| *R-CHOP and R-CHOP like*** | 35 (35%) | 8 (42%) | 27 (33%) | 0.6 |
| *DA-EPOCH-R^#^* | 18 (18%) | 3 (16%) | 15 (19%) | 1 |
| *Other**** | 5 (5%) | 0 | 5 (6%) | 0.6 |
| *Upfront ASCT* | 18 (18%) | 4 (21%) | 14 (17%) | 0.7 |
| **First response, any therapy** | | | |  |
| *ORR* | 71 (71%) | 15 (79%) | 56 (70%) | 0.5 |
| *CR* | 58 (58%) | 13 (68%) | 45 (56%) | 0.4 |
| *PR* | 13 (13%) | 2 (11%) | 11 (14%) | 1 |
| *SD/PD* | 25 (25%) | 3 (16%) | 22 (27%) | 0.3 |
| *Not evaluable* | 4 (4%) | 1 (5%) | 3 (3%) | 0.5 |

*Intensive regimens: R-CODOX-M/IVAC; GMALL-R; R-Hyper-CVAD/R-MA

**R-CHOP like: R-COMP; R-M/VACOP-B; R-mega-CHOP

***Other: R-DHAP and ASCT consolidation in 1 patient; CNS targeted treatment with R-MTX-HD-ARAC in 1 patient; palliative care in 3 patients

*^#^3 patients didn’t maintain dose intensity in DA-EPOCH-R regimen*

§CNS prophylaxis: 24 intrathecal; 8 systemic; 38 both intrathecal and systemic [*intrathecal consisted in MTX in 22 patients, MTX + ARAC in 28 patients, DepoCyte (cytarabine liposomal) in 10 patients and unknown in 2 patients*]

§§RT: 20 pts as consolidation

**Upfront ASCT (autologous stem cell transplantation):** 3 after R-CHOP/6 after DA-EPOCH-R/8 after intensive regimens/1 after R-DHAP

**Table S2: Patient and tumor characteristics according to the treatment group**

| **Treatments** | ***Intensive regimens**** | ***R-CHOP and***  ***R-CHOP like***** | ***DA-EPOCH-R^#^*** | **Significant p value** |
| --- | --- | --- | --- | --- |
| **Number of patients** | **42** | **35** | **18** |  |
| **Median age, range** | 61, 21-75 | 63, 19-80 | 59, 22-78 | ns |
| **Male sex** | 27(64%) | 20 (57%) | 12 (67%) | ns |
| **B symptoms** | 16 (38%) | 13 (37%) | 8 (44%) | ns |
| **AAS III-IV** | 39 (93%) | 28 (80%) | 12 (67%) | ^p=0.02 |
| **Elevated LDH** | 32 (76%) | 26 (74%) | 12 (67%) | ns |
| **ECOG PS ≥2** | 15 (36%) | 13 (37%) | 6 (33%) | ns |
| **Extranodal sites > 2** | 22 (52%) | 12 (34%) | 8 (44%) | ns |
| **Bone marrow involvement** | 16 (38%) | 12 (34%) | 6 (33%) | ns |
| **Bulky >= 6 cm** | 26 (62%) | 20 (57%) | 10 (56%) | ns |
| **CNS involvement** | 3 (7%) | 4 (11%) | 1 (5%) | ns |
| **IPI risk group** | | | |  |
| ***good (0)*** | 0 | 0 (%) | 1 (5%) | ns |
| ***intermediate (1-2)***  ***poor (3-4-5)*** | 10 (24%)  32 (76%) | 15 (43%)  20 (57%) | 7 (39%)  10 (56%) | ^^p=0.006 |
| **Histology** | | | |  |
| ***DLBCL NOS*** | 19 (45%) | 29(83%) | 13 (72%) | ^^^p=0.001 and p=0.05 |
| ***BCLU*** | 23 (55%) | 6 (17%) | 5 (28%) |  |
| **Cell-of-origin by Hans^§^** | | | |  |
| ***GCB*** | 27 (64%) | 23 (66%) | 14 (78%) | ns |
| ***non-GCB*** | 8 (19%) | 7 (20%) | 2 (11%) | ns |
| **Translocations** |  |  |  |  |
| ***SHL*** | 8 (19%) | 8 (23%) | 3 (17%) | ns |
| ***DHL-BCL2^§§^*** | 24 (57%) | 14 (40%) | 14 (78%) | ns |
| ***DHL-BCL6^§§^*** | 12 (29%) | 14 (40%) | 2 (11%) | ns |

*AAS: Ann Arbor stage; LDH: lactate dehydrogenase; ECOG PS: Eastern Cooperative Oncology Group performance status; CNS: central nervous system; IPI: International Prognostic Index score; DLBCL NOS: Diffuse large B cell lymphoma not otherwise specified; BCLU: “unclassifiable” aggressive B-cell lymphoma; GCB: Germinal Center B-Cell like*

*Intensive regimens: R-CODOX-M/IVAC; GMALL-R; R-Hyper-CVAD/R-MA

**R-CHOP like: R-COMP; R-M/VACOP-B; R-mega-CHOP

*^#^3 patients didn’t maintain dose intensity in DA-EPOCH-R regimen*

*^§^Not done in 15 patients*

*^ Significant difference comparing intensive regimens to DA-EPOCH R (advanced stage and ki67, the last data not shown)*

*^^ Significant difference comparing intensive regimens to R-CHOP (poor IPI)*

*^^^ Significant difference comparing intensive regimens to R-CHOP and to DA-EPOCH-R, respectively*

**Table S3: Patients characteristics and prognosis (univariate analysis)**

| **Variable** | **100 MYC-R (p*)** | **81 MYC-DHL (p*)** |
| --- | --- | --- |
| **Histology (DLBCL NOS vs BCLU)** | 0.81 | 0.68 |
| **Ki67 >90%** | 0.14 | 0.29 |
| **COO (GCB vs non-GCB)** | 0.71 | 0.69 |
| **BCL2 translocation** | 0.06 | 0.41 |
| **BCL6 translocation** | 0.77 | 0.30 |
| **Age > 60 y** | 0.82 | 0.43 |
| **Male sex** | 0.39 | 0.84 |
| **B-symptoms** | **0.05** | 0.27 |
| **ECOG PS >2** | **0.003** | **0.01** |
| **Stage III-IV** | 0.24 | 0.93 |
| **Bone marrow involvement** | **0.005** | **0.05** |
| **Bulk >= 6 cm** | 0.10 | 0.19 |
| **Extranodal sites > 2** | 0.31 | 0.46 |
| **CNS involvement** | 0.16 | 0.45 |
| **IPI poor risk** | 0.25 | 0.3 |
| **IPIaa poor risk** | **0.05** | 0.34 |
| **Elevated LDH** | **0.03** | 0.25 |
| **ASCT** | 0.09 | 0.13 |
